# The influence of transcranial alternating current stimulation (tACS) on fluid intelligence: An fMRI study

**DOI:** 10.1016/j.paid.2017.04.016

**Published:** 2017-11-01

**Authors:** A.C. Neubauer, M. Wammerl, M. Benedek, E. Jauk, N. Jaušovec

**Affiliations:** a University of Graz, Institute of Psychology, Universitätsplatz 2, 8010 Graz, Austria; b University of Maribor, Faculty of Philosophy. Koroška 160, 2000 Maribor, Slovenia

**Keywords:** Transcranial alternating current stimulation (tACS), Fluid intelligence, fMRI, Neural efficiency, Parieto-frontal integration theory (P-FIT)

## Abstract

The past decades have witnessed a huge interest in uncovering the neural bases of intelligence (e.g., Stelmack, & Houlihan, 1995; Stelmack, Knott, & Beauchamp, 2003). This study investigated the influence of transcranial alternating current stimulation (tACS) on fluid intelligence performance and corresponding brain activation. Previous findings showed that left parietal theta tACS leads to a transient increase in fluid reasoning performance. In an attempt to extend and replicate these findings, we combined theta tACS with fMRI. In a double-blind sham-controlled experiment, *N* = 20 participants worked on two intelligence tasks (matrices and paper folding) after theta tACS was applied to the left parietal cortex. Stimulation-induced brain activation changes were recorded during task processing using fMRI. Results showed that theta tACS significantly increased fluid intelligence performance when working on difficult items in the matrices test; no effect was observed for the visuo-spatial paper folding test. Whole-brain analyses showed that left parietal brain stimulation was accompanied by lower activation in task-irrelevant brain areas. Complemental ROI analyses revealed a tendency towards lower activation in the left inferior parietal cortex. These findings corroborate the functional role of left parietal theta activity in fluid reasoning and are in line with the neural efficiency hypothesis.

## 1 Introduction

Intelligence is associated with diverse relevant real-life outcomes such as educational accomplishment, occupational performance, and even health (Deary, 2012). There is a long-standing research tradition to investigate the neural bases of intelligence (e.g., Ertl & Schafer, 1969; Stelmack & Houlihan, 1995). Neurophysiological models of intelligence emphasize the role of the fronto-parietal-network. The *parieto-frontal integration* theory of intelligence (P-FIT; Jung & Haier, 2007) postulates a four-phase information processing model which highlights the importance of frontal and parietal brain areas and the associated communication patterns between those. Another important theory is the *neural efficiency hypothesis*. It posits that more intelligent people use their brain resources more efficiently as compared to less intelligent individuals in terms of lower brain activation (Haier et al., 1988) or faster neural transmission time (Stelmack, Knott, & Beauchamp, 2003). A review of relevant findings by Neubauer and Fink (2009) further suggests that neural efficiency is restricted to tasks of low to moderate task difficulty, whereas highly able individuals may even invest more cortical resources in very difficult tasks (see also, Dunst et al., 2014). More recently, there has been an increasing interest to use and extend our understanding of the brain in attempts to improve intelligence via brain stimulation (Enriquez-Geppert, Huster, & Herrmann, 2013).

### 1.1 Stimulating intelligence

Due to the social relevance of *gf*, the issue was raised whether there is a way to increase it through training or stimulation. However, recent reviews and meta-analyses come to inconsistent conclusions, either showing that intelligence scores cannot be increased through cognitive training (Haier, 2014; Melby-Lervåg, Monica, & Hulme, 2016), or finding support for the beneficial effect of systematic cognitive training (Au et al., 2015; Karbach & Verhaeghen, 2014). For example, Jaušovec and Jaušovec (2012) reported that working memory training does not only promote performance in a gf task but also changes the associated electrocortical brain activation. One particular issue of the training approach is the missing evidence for transfer effects, which is sometimes challenged by the similarity between assessment and training tasks (Shipstead, Redick, & Engle, 2012). This methodological problem can be circumvented with direct stimulation of brain activation.

Available research mostly used transcranial electrical stimulation (TES; Kuo & Nitsche, 2012), which It involves either direct (tDCS) or alternating current (tACS). TDCS manipulates neuronal activity via depolarization or hyperpolarization of cell membranes. TACS influences cortical activity via EEG frequency-specific oscillatory current. tACS leads to EEG-synchronization of neural activity in the respective frequency band, which may facilitate efficient cortical communication patterns (Zaghi, Acar, Hultgren, Boggio, & Fregni, 2010). Much research has focused on stimulation of the dorsolateral prefrontal cortex (DLPFC). For tDCS, DLPFC stimulation was associated with positive effects on cognitive processes such as language, attention, perception, executive functioning, and memory processes (Jacobson, Koslowsky, & Lavidor, 2012; Utz, Dimova, Oppenländer, & Kerkhoff, 2010). For tACS of the DLPFC, Santarnecchi, Polizzotto, Godone, Giovannelli, Feurra et al. (2013) found that gamma stimulation leads to a shortening of (correct) response latencies in a gf task.

A growing number of studies examined left-parietal stimulation effects on working memory and intelligence. Left-parietal tDCS was found to enhance performance in a verbal memory task (Jacobson, Goren, Lavidor & Levy, 2012). For tACS, Pahor and Jaušovec showed that left parietal theta tACS leads to an increase in gf performance and accompanying changes in cortical activation (Pahor & Jaušovec, 2014). The same stimulation setting also increased working memory capacity and a decreased P3 component latency, which could reflect a faster allocation of attention-related cognitive resources (Jaušovec & Jaušovec, 2014).

### 1.2 Aims of this study

Previous findings showed that left parietal theta tACS leads to a transient increase in fluid reasoning performance (Pahor & Jaušovec, 2014; Jaušovec & Jaušovec, 2014). In an attempt to replicate and extend these findings, we combined theta tACS with fMRI in a double-blind design. Available evidence indicates that left-parietal theta activity might play a causal role for gf-related demands such as executive functioning (Sauseng, Griesmayr, Freunberger, & Klimesch, 2010), working memory capacity (Postle et al., 2006). Neurophysiological stimulation effects are studied by analyzing concurrent brain activation changes with fMRI. Finally, we test the role of task difficulty as a moderating variable in the relationship between brain stimulation and brain activation (Neubauer & Fink, 2009). We expect that left parietal theta stimulation increases fluid intelligence performance (Pahor & Jaušovec, 2014) and affects brain activation in intelligence-related brain areas according to the P-FIT model (Jung & Haier, 2007). Additionally, the neural efficiency hypothesis predicts that increased intelligence performance is associated with reduced activation of brain areas that are not considered central for intelligence (Basten, Stelzel, & Fiebach, 2013; Neubauer & Fink, 2009).

## 2 Method

### 2.1 Participants

25 individuals were recruited from a pre-tested pool of participants (Jauk, Benedek, Dunst, & Neubauer, 2013). All participants were right-handed, had normal or corrected-to-normal vision, and no self-reported history of CNS-affecting drugs, mental or neurological diseases. Five participants were excluded due to excessive missing data or failure to complete both test sessions. The final sample hence consisted of 20 participants (11 women; average age = 24.85; *SD* = 3.30). The sample was kept homogeneous with respect to age (18–30 years), intelligence as measured with the intelligence-structure-battery (INSBAT; for details see Dunst et al., 2014; *M* = 106.09; *SD* = 7.34), and educational level (students) to enable a sensitive test of within-subject stimulation effects. Participants did not report any medical treatments or health problems and gave written informed consent. The study was approved by the local ethics committee.

### 2.2 Design

The study was a double-blind sham-controlled experiment. One experimenter only operated the stimulation device and had no interaction with the participant, whereas another one (who was blind to the stimulation condition) instructed participants. Verum (i.e., active) and sham (i.e., placebo) stimulation conditions varied within subjects in a counterbalanced fashion. The two sessions were separated by 28 days to control for potential influences of different phases of the menstrual cycle (Amin et al., 2006). Dependent variables were performance in the two gf tasks as well as BOLD responses in fMRI.

### 2.3 Tasks and procedure

The experiment was carried out in two sessions. Participants received a standardized instruction and then either sham or verum tACS was applied for 15 min (see below), followed by a questionnaire about intensity and duration of stimulation induced sensations. After the stimulation, the participants were led to the scanning room where they performed two intelligence tasks. The matrices task was based on the Raven's progressive matrices (RPM; Court & Raven, 1995), slightly modified to the requirements of neurophysiological investigations (Pahor & Jaušovec, 2014). The test consisted of 50 items – 18 easy (set B of the CPM, and APM items 1–3), and 32 difficult (APM items 4 to 35). The 50 test items were divided into two parallel forms each consisting of 25 items (10 easy items; 15 difficult items) which were counterbalanced between the sham/verum conditions. As in the original RPM, items were presented in a fixed sequence reflecting increasing task difficulty. Each trial of the RPM started with a jittered fixation cross period (6–10 s). Then, the item was presented for 6 s (easy items) or 10 s (difficult items) together with four response alternatives depicted below, followed by a 3 s response phase (indicated by red interrogation marks presented under the figure). During this, participants had to press one of four buttons corresponding to the four response options. Total task duration of the RPM scanner task was about 9 min. This modified RPM has been shown to correlate substantially with the WAIS-R (*r* = 0.56) (Jaušovec & Jaušovec, 2012), suggesting that content validity is not impaired by the shorter item presentation time. The cross-form consistency of the modified RPM had been proven in a larger sample (*r*_A,B_ = 0.73; Pahor & Jaušovec, 2014).

As a second intelligence measure, we used the paper folding task (PFT) from the Stanford-Binet test (Cowan et al., 2011). Participants had to judge which of the four presented figures on the right side corresponded to that one on the left side after variable steps of folding and cutting (Jaušovec & Jaušovec, 2012). Again, we used two parallel versions which consisted of 20 items each. Items were presented in a fixed quasi-randomized sequence. For analyses considering task difficulty, these items were divided in to 10 easy and 10 difficult items based on effective task performance. Presentation parameters were the same as for the RPM except that the duration of stimulus presentation was 7 s per item here, and fixation cross period varied randomly from 5 to 9 s. Total duration of the PFT was about 6 min. Again, cross-form consistency of the modified PFT was established in a previous study (*r*_A,B_ = 0.71, Pahor & Jaušovec, 2014). In both tasks (RPM and PFT) a trial was scored as solved when the correct response alternative was selected before timeout. The total duration of the experiment including instruction, stimulation, and scanning was around 65 min.

### 2.4 Electrical stimulation

We used a battery-operated stimulator system (DC-stimulator plus, Neuroconn, Ilmenau, Germany). The stimulating electrodes (5 × 7 cm) were attached to the scalp using a rubber band placed over the electrode and attached under the chin. This procedure prevented movement of the electrodes during the experiment. The electrodes were covered by saline soaked sponges, which reduced the electrode impedance. Target electrode was placed over the left parietal location (P3), and the return electrode was placed on Cz. This electrode positioning was chosen because of the key role of the left parietal cortex for gf and gf-related functions (e.g., working memory capacity; Jung & Haier, 2007; Cowan et al., 2011; Chein & Fiez, 2010), and first evidence on effects of left parietal tACS on fluid reasoning (Pahor & Jaušovec, 2014; Jaušovec & Jaušovec, 2014; Postle et al., 2006). Stimulation waveform was sinusoidal without DC offset and a 0° relative phase. The impedance level was kept below 10 kΩ throughout the entire stimulation period. The applied oscillating currents corresponded to mean theta frequency in previous studies (Jacobson, Goren, Lavidor, & Levy, 2012; 5 Hz); current intensity was 1500 μA. In verum condition, tACS was applied for 15 min. The current was ramped up and down over the first and last 15 s of stimulation. In sham condition, the procedure and stimulation parameters were the same as in the verum condition except for the duration of stimulation, which was applied for only 60 s in the beginning and then turned off. Since participants feel stimulation related sensations (e.g. itching) only in the beginning of tACS, this approach prevents individual awareness of the stimulation conditions (Nitsche et al., 2008).

### 2.5 MRI data acquisition

Imaging was performed on a 3.0-T Tim Trio system (Siemens Medical Systems, Germany) using a 32-channel head coil. BOLD-sensitive T2*-weighted functional images were acquired using a single shot gradient-echo EPI pulse sequence (TR = 2400 ms, TE = 30 ms, flip angle = 90°, slice thickness = 3.5 mm, matrix size = 68 × 68, FOV = 240 mm, 35 slices per volume). The first two volumes after each scanner pause were discarded to allow for T1 equilibration effects. Field maps were created from a double echo gradient-echo pulse sequence (31 slices, TE1 = 4.92 ms, TE2 = 7.38 ms, TR = 400 ms, slice gap = 0.9 mm, slice thickness = 3.5 mm, matrix size 68 × 68, FOV = 240 mm). Visual stimuli were presented onto a screen using the Software Presentation (Neurobehavioral Systems, Albany, CA) and viewed through a mirror attached to the head coil.

### 2.6 MRI data analysis

Functional MRI data analysis was performed using SPM 8 software (Wellcome Department of Imaging Neuroscience, London, UK). Preprocessing steps included field map correction, motion correction, slice time acquisition correction, spatial normalization to an averaged EPI template, and smoothing with a 7-mm full-width at half maximum Gaussian kernel. For each task, scans from the verum and sham fMRI sessions were modeled together in a fixed-effects model including the conditions REST (fixation epochs), VERUM, and SHAM (task performance following verum or sham stimulation, from stimulus onset to onset of response period). Motion parameters were included in the model as regressors of no interest. Task-specific brain activation was modeled with a conjunction of both stimulation and sham conditions (VERUM + SHAM > 0) against implicit baseline (Poline, Kherif, Pallier, & Penny, 2007). Stimulation-specific brain activation was modeled with the contrast of conditions (VERUM vs. SHAM). For the examination of difficulty-specific effects another model was run considering easy and difficult trials separately. At the second level, a random effects analysis was performed computing one-sample *t*-tests for the subject-specific statistical parametric maps obtained at the first level. Whole-brain results for task-specific effects (VERUM + SHAM) are reported using a conservative criterion of voxel-wise *p* < 10^−8^ (uncorrected) with cluster size of k ≥ 25. For the analysis of stimulation-specific effects (VERUM vs. SHAM), clusters are only reported if they are significant on voxel level (*p* < 0.0001, uncorrected) and exceed a minimum cluster size of 5 voxels. Finally, a region of interest (ROI) analysis was computed to determine the direction (activation or deactivation) and magnitude of changes in regions showing significant task-specific activation patterns (VERUM + SHAM) using MarsBaR 0.43 (Brett, Anton, Valabregue, & Poline, 2002). The ROIs were functionally defined based on the task-specific activation patterns (Poldrack, 2007).

## 3 Results

### 3.1 Behavioral results

The self-reported stimulation-induced sensations during the tACS sessions did not differ significantly between sham and verum tACS settings (Wilcoxon *Z*_19_ = −0.53; *ns*.).

**Fig. 1:**
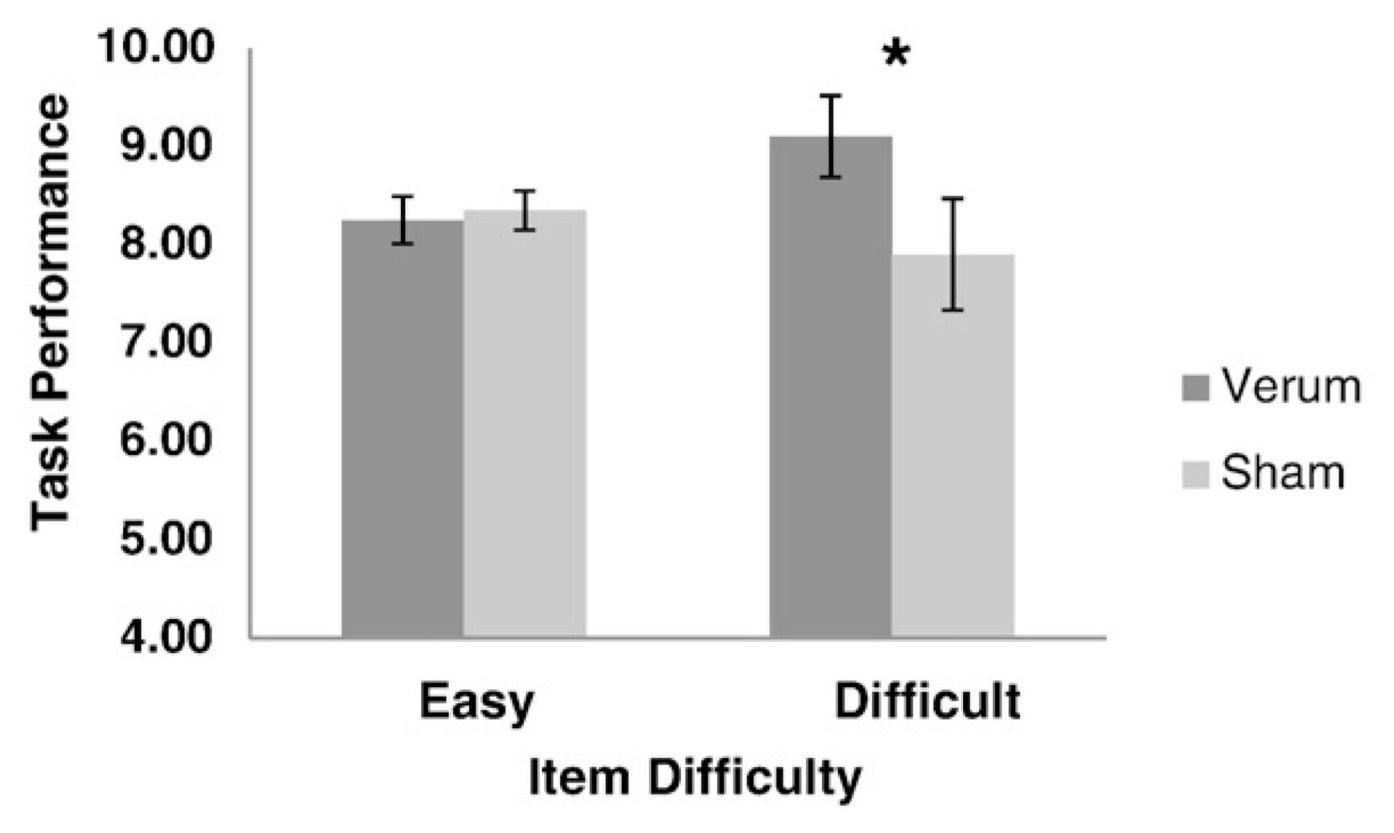
Significant interaction of task difficulty and stimulation condition (verum and sham stimulation) on task performance in the RPM task (* *p* < 0.05).

Stimulation effects on intelligence in the RPM task were analyzed with an ANOVA with the within-subject conditions STIM (sham/verum) and DIFFICULTY (easy, difficult). The interaction effect between STIM and DIFFICULTY was significant (*F*(1, 19) = 4.86, *p* < 0.05; *eta^2^*_part_ = 0.20). Post-hoc paired-sample *t*-tests showed that participants solved significantly more difficult RPM items (28.44%, *M* (*SD*) = 9.10 (1.87)) when verum tACS was applied than after sham stimulation (24.69%, *M* (*SD*) = 7.90 (2.53); *t*(19) = 2.40, *p* = 0.03, *d* = 0.53), but there was no stimulation effect for easy items (45.83%, *M* (*SD*) = 8.25 (1.07), and 46.39%, *M* (*SD*) = 8.35 (0.88) for verum and sham stimulation, respectively; *t*(19) = −0.42, *p* = 0.68; Fig. 1). Supplemental Fig. S1 shows the individual data. Performance increases following verum tACS were evident in nine of 20 individuals; seven displayed no change, four displayed (slight) decreases. Interestingly, especially those individuals with low baseline (sham) performance appear to have benefited from the stimulation.

The same GLM procedure was conducted for the PFT, but we observed no effects related to the stimulation condition or task difficulty.

### 3.2 fMRI results

#### 3.2.1 Task-specific brain activation

Task-specific brain activation in the RPM task included 13 clusters, with seven clusters showing relatively stronger activation and six clusters showing lower activation relative to baseline (Table S1). Task-specific activation was observed bilaterally in the insula, in the left superior/inferior parietal lobe, in the thalamus, the right lingual gyrus, and the postcentral gyrus. Task-specific brain activation in the PFT included 13 clusters with 8 clusters showing stronger activation and 5 clusters showing lower activation relative to implicit baseline (Table S2). Task-specific activation was observed in the middle and the left inferior occipital gyrus as well as in the left superior/inferior parietal lobe, the right insula, the right lingual gyrus, the right supramarginal gyrus, and in the right fusiform gyrus.

#### 3.2.2 Stimulation effects on brain activation

In both tasks, brain stimulation induced no relative increases but only relative decreases in brain activation (see VERUM < SHAM). In the RPM task, verum stimulation was associated with lower activation in the left middle occipital gyrus as well as in right occipital and frontal lobes (Table 1). In the PFT task, lower activation was observed bilaterally in the precuneus, the left inferior temporal gyrus, and right regions of the cerebellum. In both tasks, stimulation-induced activation changes did not overlap with the task-positive brain regions but partly overlapped with the task-negative brain regions observed for these tasks.

We further examined whether stimulation effects are different for easy and difficult items, as it was the case at the behavioral level in the RPM task. The observed activation differences were essentially the same when considering only difficult items, but no stimulation effects were observed for easy items.

#### 3.2.3 ROI analyses

Finally, we examined the effect of stimulation on brain activity in terms of signal change in the seven clusters showing increased task-specific activation (Table S1), separately for trials classified as easy or difficult. Here we observed a tendency towards a difficulty-depended stimulation effect in the inferior parietal lobe verum stimulation tended to be associated with lower activation as compared to sham stimulation for difficult items (*p* = 0.09) but not for easy items (*p* = 0.71). No stimulation effects were observed in PFT task.

## 4 Discussion

We set out to examine the effects of left-parietal theta tACS on intelligence test performance and its respective neurophysiological bases. We found that theta tACS can enhance performance in a gf task (as measured by Raven's Progressive Matrices) for difficult items; no stimulation effect was found for easy RPM items, or the PFT. The stimulation effect was accompanied by distinct brain activation changes. Now, we will discuss reasons and implications of these findings.

This study replicates previous research showing that left parietal theta tACS leads to increased task-specific reasoning performance (Pahor & Jaušovec, 2014). It is also in line with findings that left parietal theta tACS increased working memory capacity (Jaušovec & Jaušovec, 2014), as working memory is a central executive function underlying fluid intelligence (Benedek, Jauk, Sommer, Arendasy, & Neubauer, 2014). We presume that theta frequency reflects a general cognitive control mechanism, which might be of general importance for gf performance (Sauseng et al., 2010).

Regarding the neurophysiological stimulation effects, left parietal theta stimulation was accompanied by lower brain activation in the right frontal lobe and bilaterally in the occipital lobe for the RPM, and in the precuneus, left inferior temporal gyrus, and right cerebellum in the PFT task. The missing overlap of stimulation-induced brain activation changes across tasks can be seen to corroborate the task-specificity of the stimulation effects. Furthermore, independent of the stimulation condition, there was little overlap in task-specific activation patterns (e.g. *inferior* parietal lobule, insula; Table S1; Table S2), which supports the notion that each of the tasks addresses distinct facets of intelligence performance.

Notably, stimulation-induced decreases in brain activation primarily concerned brain areas that were not part of task-specific brain activation patterns (i.e., task-positive areas), but rather overlapped with task-negative areas (e.g., precuneus). This finding parallels research on neural efficiency: Studies have shown that higher intelligence is related to lower brain activation, particularly in task-negative brain regions (Neubauer & Fink, 2009; Basten et al., 2013). Our findings could thus be tentatively interpreted in terms of induced neural efficiency by means of tACS.

A closer look suggests that activation decreases might also be related to task difficulty. Complemental analyses showed that stimulation-related differences in brain activation were only apparent when individuals worked on difficult, but not on easy items (mirroring the behavioral stimulation effects). The stronger deactivation following stimulation during difficult tasks might indicate an efficient downregulation of irrelevant cortical activity during phases of increased cognitive load.

Interestingly, we did not observe stimulation-dependent brain activation increases in task-positive brain regions or parietal and frontal areas as predicted by the P-FIT (Jung & Haier, 2007). In contrast, ROI analyses revealed a weak tendency towards a stimulation-induced reduction of brain activation in the left inferior parietal cortex. While the left inferior parietal cortex is part of the task-positive network, it was also the stimulation location. Hence, theta stimulation may not necessarily translate to increased brain activation, but may even induce slight relative deactivation; at least at the stimulation site. However, these stimulation-specific effects did not survive FWE-correction, why they should be seen as first exploratory evidence for a potential impact of theta-tACS on the hemodynamic function measured through fMRI.

Our findings add to evidence reported by Jaušovec and Jaušovec (2014), who observed a decreased P3 latency and increased theta power at left parietal brain regions after theta tACS was applied on the left parietal cortex (cf., Beauchamp & Stelmack, 2006). Moreover, Pahor and Jaušovec (2014) showed that parietal theta tACS was associated with a frontal theta power increase. How can these previous EEG findings be reconciled with the fMRI evidence? Scheeringa et al. (2009) showed that, during performance of a working memory task, increases in frontal theta power were correlated with BOLD decreases in regions that together form the default mode network. Thus, our finding of a deactivation of brain regions that are not considered essential for gf performance is generally consistent with the observed frontal theta power increase in a previous study (Pahor & Jaušovec, 2014), and it seems that higher theta power goes along with lower BOLD in independent brain regions. Altogether, our study suggests that the main mechanism underlying theta tACS-stimulation effects can be seen in brain activation decreases of task-irrelevant brain regions rather than increases in task-relevant regions, which is in line with the neural efficiency hypothesis. This finding is potentially consistent with the notion that intelligence is not associated with faster neural transmission at task-relevant regions (Stelmack et al., 2003).

It should be acknowledged, however, that the current findings can only be interpreted in terms of a transient increase in the performance on a specific fluid intelligence task, which can be seen as an enhancement of a subfactor (namely fluid reasoning) rather than a general “intelligence boost”. Note also that stimulation effects were restricted to task performance on Raven items of higher difficulty. This includes the behavioral and brain activation effects and is consistent with a previous study (Pahor & Jaušovec, 2014). But why are findings specifically observed for difficult but not for easy Raven items? Easy items had item difficulties ranging between 0.76 and 1 indicating that they were solved by most of the participants. Hence, easy items may have less discriminatory power than more difficult items to discern between differences in reasoning performance. Also, complemental single-subject analyses showed that individuals with low baseline performance benefit the most from tACS, which has direct implications for the differential use of tACS and will hopefully stimulate future research.

Finally, stimulation effects were specific to the matrices task in this study. A possible explanation could be that the RPM and the PFT focus on different facets of intelligence. Although the PFT is commonly considered a fluid intelligence task (e.g., Nusbaum & Silvia, 2011), it can also be seen to have a strong visual-spatial focus. Recent research showed that visual-spatial abilities are predominantly represented via right-hemispheric activation (Zacks, 2008), whereas fluid reasoning is mainly represented through left-hemispheric (Barbey et al., 2012) or bilateral activation patterns (Gray, Chabris, & Braver, 2003). A left parietal stimulation hence could have more effect on cognitive processes that are generally left-lateralized. Also, the participants performed the matrices task first, so it is possible that the power of the stimulation effect had already decreased when participants worked on the PFT.

In conclusion, this study was the first to explore the neurophysiological basis of fluid intelligence via the combination of tACS and fMRI. Left parietal theta tACS was found to moderately increase performance in a fluid reasoning task when working on difficult items, and this was accompanied by deactivation of task-irrelevant brain regions. For future research, it would be exciting to study tACS effects on additional direct indices of brain functioning like neural transmission time (e.g., Stelmack et al., 2003).

## Supplementary Material

Supplementary data to this article can be found online at http://dx.doi.org/10.1016/j.paid.2017.04.016.

Supplementary material

## Figures and Tables

**Table 1:** Stimulation-induced brain activation changes for the two intelligence tasks (RPM and PFT).

Contrast	MNI peak coordinate	k	t	Brain area
RPM				
VERUM > SHAM	−	−	−	−
VERUM < SHAM	34 –60 –5	6	4.39	Occipital lobe (right)
	38 –21 27	6	4.28	Frontal lobe (right)
	–43 –81 –1	5	4.05	Middle occipital gyrus (left)
PFT				
VERUM > SHAM	−	−	−	−
VERUM < SHAM	10–53 34	16	4.68	Precuneus (right)
	–50 –14 – 26	7	4.56	Inferior temporal gyrus (left)
	–12 –49 34	13	4.43	Precuneus (left)
	24–84 –40	7	3.97	Cerebellum (right)

Notes. *p* < 0.0001; k ≥ 5.
