# Supplementary material for: The influence of transcranial alternating current stimulation (tACS) on fluid intelligence: An fMRI study
Source: Pers Individ Dif. Author manuscript; Available in PMC 2017 Nov 23. (PMC5700801; doi:10.1016/j.paid.2017.04.016)
Supplement: Supplementary material [file NIHMS74968-supplement-Supplementary_material.docx]

**Supplementary material**

Table S1.

*Task-specific brain activation in the matrices task (RPM).*

|  | *MNI Peak Coordinate* | *k* | *t* | *Brain Area* |
| --- | --- | --- | --- | --- |
| VERUM + SHAM > 0 | 13 -91 -8 | 857 | 19.15 | Lingual Gyrus (right) |
|  | -47 -35 48 | 58 | 18.46 | Inferior Parietal Lobe (left) |
|  | 31 25 -5 | 48 | 18.15 | Insula (right) |
|  | -29 25 -5 | 29 | 15.65 | Insula (left) |
|  | 48 -28 45 | 170 | 15.56 | Postcentral Gyrus (right) |
|  | -19 -7 10 | 42 | 14.40 | Thalamus |
|  | -26 -63 41 | 81 | 13.30 | Superior Parietal Lobe (left) |
|  | | | | |
| VERUM + SHAM < 0 | -8 -56 -20 | 114 | 14.77 | Precuneus (left) |
|  | -19 -46 6 | 25 | 14.22 | Precuneus (left) |
|  | -1 -25 45 | 45 | 12.92 | Middle Cingulum (medial) |
|  | -8 46 -1 | 26 | 12.54 | Anterior Cingulum (left) |
|  | -1 56 17 | 42 | 11.90 | Frontal Lobe (medial) |
|  | 41 -11 17 | 39 | 11.65 | Rolandic Operculum (right) |

*Notes.* *p* < 10^-8^ (uncorr.), *k* ≥ 25

Table S2.

*Task-specific brain activation for the paper folding task (PFT).*

| *Contrast* | *MNI Peak Coordinate* | *k* | *t* | *Brain Area* |
| --- | --- | --- | --- | --- |
| VERUM + SHAM > 0 | 17 -91 -8 | 155 | 17.29 | Lingual Gyrus (right) |
|  | -29 -91 10 | 36 | 16.28 | Middle Occipital Gyrus (left) |
|  | -47 -35 45 | 36 | 14.81 | Inferior Parietal Lobe (left) |
|  | -22 -67 48 | 45 | 14.01 | Superior Parietal Lobe (left) |
|  | 38 -39 45 | 132 | 14.01 | Supramarginal Gyrus (right) |
|  | 31 25 -5 | 25 | 13.75 | Insula (right) |
|  | -29 -88 -12 | 123 | 13.00 | Inferior Occipital Gyrus (left) |
|  | 31 -42 -19 | 34 | 11.49 | Fusiform Gyrus (right) |
|  |  |  |  |  |
| VERUM + SHAM < 0 | -5 -49 34 | 166 | 16.01 | Precuneus (left) |
|  | -12 53 34 | 95 | 14.91 | Superior Frontal Gyrus (left) |
|  | -1 -21 45 | 42 | 13.69 | Fusiform Gyrus (medial) |
|  | -64 -49 24 | 39 | 13.44 | Supramarginal Gyrus (left) |
|  | -57 -21 -15 | 25 | 11.87 | Middle Temporal Gyrus (left) |

*Notes.* *p* <10^-8^ (uncorr.), *k* ≥ 25

*Supplemental Figure S1.* Individual data of Raven performance (easy vs. difficult items) under verum and sham stimulation conditions.
